# Occurrence and Genotypic Characterization of Selected Multidrug-resistant ESKAPE-E Pathogens Isolated from Integrated Smallholder Fresh Produce Farms

**DOI:** 10.1016/j.jfp.2025.100543

**Published:** 2025-06-23

**Authors:** Sheldon Viviers, Loandi Richter-Mouton, Jonathan Featherston, Lise Korsten

**Affiliations:** 1 Department of Plant and Soil Sciences, University of Pretoria, Hatfield, Pretoria 0028, South Africa; 2 Department of Science and Innovation-National Research Foundation Centre of Excellence Food Security, Pretoria 0001, South Africa; 3 National Institute for Communicable Diseases, Sequencing Core Facility, Sandringham 2131, South Africa

**Keywords:** Antimicrobial resistance, ESKAPE-E, Food safety, Water-soil-plant-nexus, WGS

## Abstract

Antimicrobial resistance (AMR) ranks among the top ten global public health threats, with extended-spectrum β-lactamase (ESBL)-producing Enterobacterales and *Pseudomonas aeruginosa* emerging as multidrug-resistant (MDR) priority pathogens. This study aimed to evaluate the presence and characteristics (phenotypic and genotypic AMR profiles) of ESBL/AmpC-producing Enterobacterales and *P. aeruginosa* isolates obtained from the water-soil-plant nexus on smallholder fresh produce farms in South Africa. Samples of irrigation water (*n* = 44), soil (*n* = 85), and fresh produce (*n* = 95) were collected from six smallholder farms. Potential MDR bacteria were isolated using selective enrichment, chromogenic media, and identified with MALDI-TOF MS. Antimicrobial resistance was assessed via double-disk diffusion against 13 different antibiotic classes, while 20 selected isolates were genotypically characterized using whole-genome sequencing (WGS) (Illumina MiSeq). ESBL/AmpC-producing isolates were detected in 17% of samples (38/224), primarily from fresh produce (54%), followed by soil (31%) and water (15%). The dominant Enterobacterales species identified from all three matrices included *Enterobacter* spp. (26%), *Klebsiella* spp. (26%), *Serratia* spp. (17%), and *Escherichia coli* (9%). Phenotypically, 47% and 43% of the isolates were classified as ESBL and AmpC-producers, respectively, with resistance to beta-lactams, aminoglycosides, and glycylcyclines. Notably, WGS analysis indicated the presence of clinically significant AMR and virulence genes among isolates from the environment, particularly those associated with mobile genetic elements. The results from the study add to the limited national and international knowledge of AMR prevalence in the smallholder agricultural landscape, needed for the introduction of appropriate intervention strategies and future risk assessment of the informal supply chain.

## Highlights

• ESBL/AmpC-producing isolates were detected in 17% of all samples (38/224).
• MDR was observed for all isolates, barring one *S. enterica* strain (baby carrots).
• Notable ARGs were detected, including *mcr-9,* encoding colistin resistance.
• Across various farm environments, a wide range of bacterial species shared ARGs.
• Genes linked to MGEs suggest potential transfers between environmental microbiota.

Antimicrobial resistance (AMR) is one of the most pressing global health challenges (World Health Organization [WHO], 2024). The growing occurrence of multidrug resistant (MDR) pathogens has led to compromised healthcare treatment options, rendering many ineffective (Koutsoumanis et al., 2021; Rahman et al., 2022). According to the European Antimicrobial Resistance Surveillance Network (EARS-Net), more than 800,000 infections and over 35,000 deaths annually are attributed to antibiotic-resistant bacteria (ARB) across Europe (European Centre for Disease Prevention and Control [ECDC], 2022). In the European Union, the most prevalent AMR bacterial species reported in clinical sectors in 2021 was *Escherichia coli* with approximately 40% prevalence, followed by *Klebsiella pneumoniae*, *Pseudomonas aeruginosa*, and *Enterococcus faecalis* (EARS-Net, 2022). The United States Centers For Disease Control And Prevention (CDC) in 2024 reported an increased burden of carbapenem-resistant Enterobacterales (CRE), ESBL-producing Enterobacterales, and MDR *Pseudomonas aeruginosa* among others from 2021 to 2022 in healthcare settings across the United States of America.

In sub-Saharan Africa, *E. coli, Streptococcus pneumoniae, K. pneumoniae*, and *P. aeruginosa* are reported as the primary MDR pathogens contributing to fatalities (Murray et al., 2022). Some of the pathogens mentioned, like *K. pneumoniae*, *P. aeruginosa*, and *E. coli*, are part of the ESKAPEE group, known for their ability to “escape” the effects of common antibiotics (de Oliveira et al., 2020). This ability presents serious challenges in both healthcare and environmental settings, where these bacteria persist (Denissen et al., 2022). Over the past decade, there has been a growing focus on the role of environmental transmission of AMR, prompting calls for a coordinated, One Health approach (WHO, 2015; Koutsoumanis et al., 2021). A recent review by Denissen et al. (2022) highlighted the environment as a reservoir for ARB, illustrating how antibiotic-resistant ESKAPEE pathogens persist outside clinical settings, particularly in water sources and within the food chain.

Antimicrobial resistance, detected across diverse sources, remains a critical challenge within the One Health framework, given its widespread impact on human health, animal welfare, and the environment (Taggar et al., 2020; Wang et al., 2021, Sung et al., 2022). The recent United Nations General Assembly High-Level Meeting underscored this issue, emphasizing the need for global, coordinated action across sectors to mitigate the escalating burden of AMR (World Health Organization (WHO), 2024a, World Health Organization, 2024b). The recognition of AMR as an environmental issue at the General Assembly further supports adopting a One Health approach to address AMR across all sectors (World Health Organization (WHO), 2024a, World Health Organization, 2024b). In recent years, the spread of Gram-negative bacteria that produce extended-spectrum β-lactamases (ESBLs) and exhibit resistance to third-generation cephalosporins (3GC), along with carbapenems, has been increasingly reported (Centers for Disease Control and Prevention, 2024, World Health Organization (WHO), 2024a, World Health Organization, 2024b). Antibiotic−resistant bacteria (ARB) of clinical importance, found not only in healthcare settings but also in agricultural environments, are often treated using last-resort medications (Jiménez-Belenguer et al., 2023; Richter et al., 2023). They also share several critical biological traits, including the ability to adapt and thrive in the One Health environment (including healthcare and agriculture) (Miller and Arias, 2024, Oyenuga et al., 2024). Moreover, they exhibit a wide array of mechanisms for acquiring resistance genes and facilitating the global spread of high-risk clones (Miller and Arias, 2024, Oyenuga et al., 2024).

Surveillance of ARB has become increasingly common in food safety research, reflecting a growing awareness of AMR risks in food systems (Ben Said et al., 2016, Ifedinezi et al., 2024). However, monitoring ARB within agricultural environments remains challenging, as varying agricultural practices and environmental conditions exist which can influence the persistence and spread of ARB (Denissen et al., 2022). This challenge is especially evident in low- and middle-income countries, where diverse environmental conditions and limited resources further complicate consistent and effective surveillance systems (Mafiz et al., 2018). In South Africa (SA), the prevalence of ESBL-producing bacteria in commercial fresh produce farms has been documented (Richter et al., 2020). However, there is limited information on the prevalence of these pathogens in smallholder farms (Beharielal et al., 2018, Kgoale et al., 2024).

Smallholder farming is defined as the cultivation of small areas of land (<10 ha) by families who predominantly rely on their own labor (Lowder et al., 2016) and supply a large majority (80%) of the South African population with fresh produce, even though unregulated (Baloyi et al., 2022). The presence of ARB within the water-soil–plant nexus of these farms could thus potentially pose a risk to consumers. Addressing this knowledge gap is essential, as policies increasingly promote smallholder farming to enhance household food security and reduce poverty (StatsSA, 2021; Savary et al., 2022). This study aimed to determine the occurrence, identity, and antimicrobial resistance profiles of ESBL- and AmpC-producing Enterobacterales and *P. aeruginosa*, including selected ESKAPE-E pathogens throughout the water–soil–plant nexus from highly diversified smallholder farms in South Africa. In addition, the study aimed to genotypically characterize a selected number of clinically significant isolates using whole-genome sequencing.

## Materials and methods

### Sample collection and processing

Environmental sources, including irrigation water, soil, and fresh produce, were sampled from six different smallholder farms (A–F), i.e. aquaculture, integrated, organic, and conventional farming, in the Gauteng, North West, and Limpopo Provinces of SA (Supplementary Table S1; Viviers et al., 2024). Sampling was carried out on each farm during a single visit in 2022, with Farms A to D sampled during the dry season (June–September) and Farms E and F during the wet season (October–December). A total of 224 samples, including 44 water, 85 soil, and 95 fresh produce, were collected from the six smallholder farms (Supplementary Table S1). Water samples were collected at various points in the irrigation system, where accessible, comprising 22 from boreholes, 11 from rivers, and 11 from municipal water sources. The one-liter water samples were filtered through nitrocellulose membranes (0.45 µm pore size, Sartorius, Gottingen, Germany), subsequently placed in 50 mL buffered peptone water (BPW) (Merck, Johannesburg, SA) and incubated at 37 °C for further enrichment of presumptive foodborne pathogens. The kidney dialysis filter membranes, used for the 100 L water samples, were back-flushed with 2.5 L Tween-80 dH20 and the subsequent back-flushed liquid filtered through 0.45 µm nitrocellulose membranes similar to the 1 L water samples (FDA, 2021). Soil samples (25 g each) were mixed with 225 mL BPW and incubated at 37 °C to detect potential foodborne pathogens. Leafy vegetable samples (50 g each) were placed in 200 mL BPW at a 1:4 ratio, while other whole-vegetable types (150 g each) were placed in 150 mL BPW at a 1:1 ratio, with all incubated at 37 °C for potential pathogen detection (Richter et al., 2019).

### Isolation and identification of presumptive ESBL- and AmpC-producing Enterobacterales and *Pseudomonas aeruginosa* from environmental samples

After an initial four–hour incubation at 37 °C, 1 mL aliquots of each BPW/sample mixture were transferred into 9 mL of Enterobacteriaceae Enrichment (EE) broth (Oxoid Ltd, Johannesburg) and incubated at 30 °C for 24 h for selective enrichment. Each enriched sample was plated onto ChromID ESBL agar plates (BioMerieux, Johannesburg), using the streak plate method, and incubated at 30 °C for 24 h (Blaak et al., 2014). All presumptive positive ESBL-/AmpC-producing Enterobacterales colonies were isolated from the selective chromogenic media and subsequently purified. The purified isolates were then streaked onto nutrient agar (Millipore, Merck, Johannesburg) and incubated at 37 °C for 24 h before confirmation of identities using matrix-assisted laser-desorption ionization time-of-flight (MALDI-TOF) analysis as previously described (Viviers et al., 2024).

### Phenotypic antibiotic susceptibility testing

Antibiotic susceptibility was assessed using the Kirby Bauer disk diffusion technique (Clinical and Laboratory Standard Institute [CLSI], 2024). Briefly, isolates were cultured in 9 mL Tryptone Soy Broth (TSB) (Merck) and incubated for 24 h at 37 °C. Of each TSB sample, 100 μl was cultured in 9 mL of Brain Heart Infusion (BHI) broth (Merck) and incubated at 37 °C for 24 h. A 120 μl aliquot was then plated onto Mueller-Hinton (MH) agar plates (ThermoFisher Scientific, Johannesburg, SA), in duplicate and incubated at 37 °C for 24 h. The double disk synergy test (DDST) was used to confirm ESBL production: using cefpodoxime (10 μg), cefotaxime (30 μg), and ceftazidime (30 μg) in combination with, and without clavulanic acid (10 μg) (Mast Diagnostics, Randburg, SA) (European Committee on Antimicrobial Susceptibility Testing [EUCAST], 2024). Lastly, AmpC production was verified using an AmpC detection set (Mast Diagnostics) (European Committee on Antimicrobial Susceptibility Testing, 2024, Clinical Laboratory Standard Institute [CLSI], 2020). Additional antibiotics used for resistance profiling of the isolates are listed in Table 1, with the zone diameters measured and compared per CLSI (2020) and EUCAST (2024) guidelines using the BioNumerics software version 7.6 (Applied Mathematics, Pretoria, SA). Isolates resistant to three or more different antibiotic classes were classified as multidrug-resistant (MDR).

**Table 1:** Antibiotics, grouped by class, for determining bacterial resistance profiles in this study

Antibiotic classes		Antibiotic	Concentration	Abbreviation
Aminoglycosides		Gentamicin	10 μg	GM10C
		Streptomycin	10 μg	S10C
		Aztreonam	30 μg	ATM30C

Chloramphenicol		Chloramphenicol	30 μg	C30C
Beta-lactams	Penicillins	Ampicillin	10 μg	AP10C
		Amoxicillin	10 μg	A10C
	Carbapenems	Imipenem	10 μg	IMI10C
		Meropenem	10 μg	MEM10C
	Cephalosporins II	Cefoxitin	30 μg	FOX30C
		Cefpodoxime	10 μg	CPD10C
	Cephalosporins III	Ceftazidime	30 μg	CTX30C
		Cefotaxime	30 μg	CAZ30C
	Cephalosporins IV	Cefepime	30 μg	CPM30C

Fluoroquinolones		Norfloxacin	10 μg	NOR10C
		Ciprofloxacin	15 μg	CIP15C

Glycylcyclines		Tigecycline	15 μg	TGC15C

Macrolides		Azithromycin	15 μg	ATH15C
		Erythromycin	15 μg	ERY15C

Quinolones		Nalidixic acid	30 μg	NA30C
Sulfonamides		Trimethoprin-sulfamethoxazole/ cotrimoxazole	1.25 μg/23.75 μg	TS25C
Tetracyclines		Tetracycline	30 μg	T30C

### Genomic DNA extraction, whole genome sequencing, and analysis

**DNA extraction.** The genomic DNA of 20 selected isolates (10 Enterobacterales and 10 *Pseudomonas aeruginosa* isolates) was extracted using the DNeasy PowerSoil kit (Qiagen, Johannesburg, SA) according to the manufacturer’s instructions (Table 2). After gDNA extraction, the concentration and quality of the extracted DNA were determined using the Qubit dsDNA Broad Range Assay (Life Technologies, Johannesburg) and Nanodrop 2000 (ThermoScientific), respectively.

**Table 2:** Isolates selected for whole genome sequence analysis from the six different smallholder farm environments in South Africa

Strain (*UPMP)	Organism identity	Source	Farm	System/scenario
*2457	*Serratia marcescens*	Borehole water	B	Integrated
*2464	*Pseudomonas aeruginosa*	Borehole water	C	Organic
*2388	*Escherichia coli*	River water	D	Conventional/river
*2468	*Escherichia coli*	Baby carrots	D	Conventional/river
*2474	*Enterobacter cloacae*	Leeks	D	Conventional/river
*2456	*Salmonella enterica.*	Baby carrots	D	Conventional/river
*2469	*Pseudomonas aeruginosa*	Baby carrots	D	Conventional/river
*2470	*Pseudomonas aeruginosa*	Spring onions	D	Conventional/river
*2472	*Pseudomonas aeruginosa*	Leeks	D	Conventional/river
*2475	*Pseudomonas aeruginosa*	Rocket	D	Conventional/river
*2476	*Pseudomonas aeruginosa*	Rocket	D	Conventional/river
*2478	*Pseudomonas aeruginosa*	Soil	D	Conventional/river
*2488	*Pseudomonas aeruginosa*	Irrigation water	E	Conventional/dam
*2439	*Escherichia coli*	Soil	F	Conventional/municipal
*2492	*Enterobacter cloacae*	Soil	F	Conventional/municipal
*2496	*Klebsiella pneumoniae*	Spinach	F	Conventional/municipal
*2497	*Klebsiella pneumoniae*	Bell pepper	F	Conventional/municipal
*2499	*Klebsiella pneumoniae*	Onions	F	Conventional/municipal
*2502	*Pseudomonas aeruginosa*	Onions	F	Conventional/municipal
*2505	*Pseudomonas aeruginosa*	Municipal water	F	Conventional/municipal

**Whole genome sequencing and analysis.** Multiplexed paired-end libraries (2 × 300 bp) were generated utilizing the Nextera XT DNA sample preparation kit (Illumina, San Diego, CA, USA). Sequencing was conducted on an Illumina MiSeq platform with a coverage depth of 100× at the National Institute of Communicable Diseases (NICD) Sequencing Core Facility in SA. The initial raw reads underwent quality assessment, trimming, and *de novo* assembly into reads/contigs utilizing CLC Genomics Workbench version 10 (CLC, Bio-QIAGEN, Aarhus, Denmark). Subsequently, the *de novo* assembled reads/contigs were submitted to GenBank for annotation using the NCBI prokaryotic genome annotation pipeline server (https://www.ncbi.nlm.nih.gov/genome/annotation_prok/). The sequence reads were also processed to remove poor-quality regions (<Q20) and adapter sequences using Trim Galore. The trimmed reads were then aligned to their respective reference genomes using Bowtie2 (v2.4.2) in “very-sensitive” mode to maximize alignment accuracy. Postalignment processing was performed using SAMtools (v1.18): alignment files were converted from SAM to BAM format, sorted, and indexed for downstream analysis. Mapped reads were subsequently extracted from the sorted BAM files using SAMToFastq (v3.0). Finally, a reference-guided assembly was extracted using a Bayesian consensus mode in SAMtools (v1.18).

Genotypic characterization followed by identifying antibiotic resistance genes (ARGs), multilocus sequence types (MLST), pathogen likelihood, and mobile genetic elements (MGEs), by searching the ResFinder, MLST, PathogenFinder, and MGE databases at the Centre for Genomic Epidemiology (CGE) (https://cge.cbs.dtu.dk/services/), respectively (Larsen et al., 2012, Cosentino et al., 2013, Bortolaia et al., 2020, Johansson et al., 2021). Default parameters were considered for all of the software used unless otherwise indicated. Additionally, ABRicate version 1.0.1 (https://github.com/tseemann/abricate) was used for AMR gene presence analysis (Seeman, 2016, Feldgarden et al., 2019*,* retrieved from https://github.com/tseemann/abricate). Enterobacterales and *P. aeruginosa* virulence factors and serotypes were identified using VirulenceFinder, SeroTypeFinder, and PAst on the CGE platforms (Thrane et al., 2016, Malberg-Tetzschner et al., 2020). Additionally, *Klebsiella pneumoniae* isolates were serotyped based on the capsule polysaccharides (K-type) using Kaptive Web (Wick et al., 2018).

## Results

### Prevalence of presumptive extended-spectrum beta-lactamase /AmpC-producing Enterobacterales and *Pseudomonas aeruginosa* isolates from the water-soil-plant nexus

**Figure 1:**
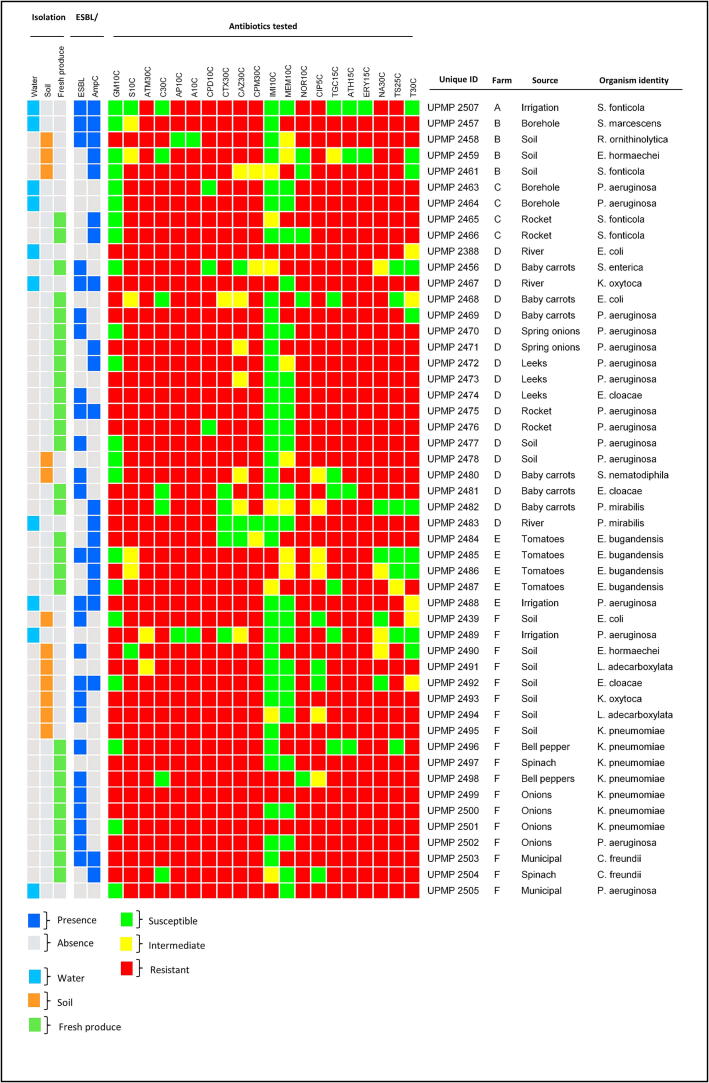
Extended-spectrum and AmpC β-lactamase producing Enterobacterales (including *Serratia fonticola* and *Serratia marcescens*, *Enterobacter hormaechei, Enterobacter cloacae* and *Enterobacter bugandensis, Escherichia coli, Klebsiella pneumoniae* and *Klebsiella oxytoca*, *Leclercia adecarboxylata,* and *Citrobacter freundii*, and *Pseudomonas aeruginosa* isolated from the environment of diverse smallholder farms.

A total of 35 Enterobacterales isolates, including *Enterobacter* spp. (26%), *Klebsiella* spp. (26%), *Serratia* spp. (17%), *E. coli* (9%), *Proteus* spp. (6%), *Citrobacter* spp. (6%), *Leclercia* spp. (6%), *Salmonella enterica*, and *Raoultella ornithinolytica*, were phenotypically characterized (Fig. 1). The majority were sourced from fresh produce (19/35, 54%), followed by soil (11/35, 31%) and water (5/35, 15%) (Fig. 1). In contrast, the 15 *P. aeruginos*a isolates were from fresh produce (8/15, 53%), followed by water (5/15, 33%) and soil (2/15, 14%) (Fig. 1). From the six different smallholder farms, Farm D, using river water for irrigation, had the highest diversity of genera (8/11) including ESBL-producing *S. marcescens* and *E. coli*. Farm F, using municipal water for irrigation and chicken manure as a soil amendment, had the second-highest diversity with six different genera. The occurrence of the selected ESKAPE-E strains from the different water source types was the highest for borehole and river water (four isolates each), followed by municipal (three isolates) and dam (one isolate) water. Interestingly, the highest occurrence of isolates from soil was observed for Farms F (seven isolates) and E (four isolates) where animal manure was used as a soil amendment, compared to Farms C and E (no isolates) where organic compost heaps were used (Fig. 1).

### Antimicrobial resistance profiles of selected extended-spectrum β-lactamase (ESBL)- and/or AmpC-producing ESKAPE-E isolates

A total of 19/35 (54%) isolates tested positive for ESBL production and 18/35 (51%) for AmpC production using the double-disk synergy test (DDST). (Fig. 1). Among the *P. aeruginosa* isolates, 5/15 (33%) were ESBL-positive and 4/15 (27%) AmpC-positive (Fig. 1). Of the 19 ESBL-producing Enterobacterales, ten (50%) were from fresh produce, followed by soil (5/19) and water (4/19) (Fig. 1). Similarly, most AmpC-producers were from fresh produce (9/18; 50%), with soil 5/18 (28%) and water 4/18 (22%) as secondary sources (Fig. 1). All the Enterobacterales isolates were resistant to aminoglycosides, penicillins, and glycylcyclines, with high resistance to fluoroquinolones (*n* = 34; 97%), macrolides (*n* = 33; 94%), and lastly quinolones, and cephalosporins (*n* = 32; 92% each) (Fig. 1). Likewise, all *P. aeruginosa* isolates were resistant to aminoglycosides, glycylcyclines, and fluoroquinolones/quinolones, with 93% resistant to penicillins, sulfonamides, and tetracyclines (Fig. 1). Lastly, similar AMR profiles were found across isolates from water, soil, and fresh produce. All 51 isolates resisted aztreonam (an aminoglycoside) only, followed by cefepime (one susceptible isolate), streptomycin, ampicillin, amoxicillin, and erythromycin (with two susceptible isolates each), ceftazidime (three susceptible isolates), norfloxacin, ciprofloxacin, azithromycin, and nalidixic acid (with four susceptible isolates each) (Fig. 1). The most effective antimicrobials, namely imipenem, meropenem, and gentamycin, inhibited the growth of 36 (71%), 25 (49%), and 22 (43%) isolates, respectively (Fig. 1). Notably, multidrug resistance was observed in all of the isolates (*n* = 51). (Fig. 1).

### Genotypic characterization of selected ESKAPE-E pathogens

The 20 isolates selected for whole genome sequencing spanned six genera: *Serratia, Pseudomonas, Escherichia, Enterobacter, Salmonella,* and *Klebsiella* (Table 2). These isolates were selected based on the isolation source (water, soil, or fresh produce) and respective phenotypic resistance profiles (Fig. 1). Notably, several isolates belonged to the ESKAPE-E pathogen group published by the WHO in 2024, including *P. aeruginosa* (*n* = 10), *Enterobacter cloacae* (*n* = 2), *Klebsiella pneumoniae* (*n* = 3), and *E. coli* (*n* = 3), which separate known bacterial species into different priority pathogen groups depending on their clinical significance. The *Salmonella enteric*a isolate was included in the analysis as an isolated potential foodborne pathogen (Kgoale et al., 2024).

**Figure 2:**
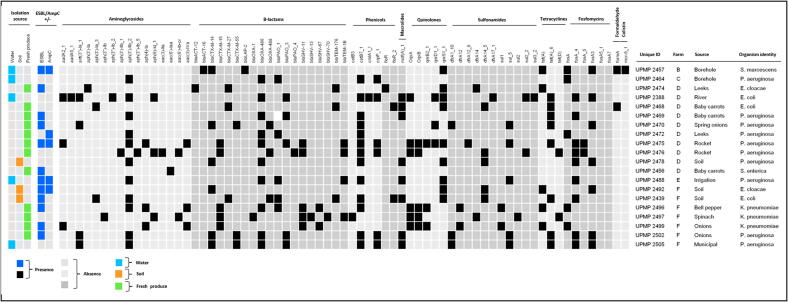
Antimicrobial resistance genes detected, using whole-genome sequencing and ResFinder, in ten Enterobacterales and *Pseudomonas aeruginosa* strains each, isolated from smallholder farming environments in South Africa.

**Detection of antimicrobial resistance genes.** Except for a single isolate (*S. enterica*), all carried at least one carbapenem and/or cephalosporin resistance genetic determinant (Fig. 2) (Supplementary Table S2). The most prevalent β-lactamase genes were *blaOXA-like* (70%, 14/20) and *blaPAO-like* (55%, 11/20) (Fig. 2). Furthermore, the *catB* gene and its variants encoding chloramphenicol resistance were detected in 70% (14/20) of the isolates, while the *sul* (sulfonamide resistance) and *tet(A)* (tetracycline resistance), genes were present in 55% (11/20) and 65% (13/20), respectively (Fig. 2). Across all the isolates, only one macrolide-resistance gene (*mdf(A)_1*) was present, detected in 40% (8/20) of the isolates (Fig. 2) (Supplementary Table S2). Notably, the *S. marcescens* strain from borehole water harbored the clinically significant colistin resistance gene, *mcr-9* (Fig. 2). Additionally, two isolates (*S. marcescens* from borehole water and *E. coli* from baby carrots) harbored the formaldehyde resistance gene, *formA* (Fig. 2). A high diversity of aminoglycosides and beta-lactam resistance genes were observed, with 16 and 19 different genetic determinants, respectively (Fig. 2).

**Figure 3:**
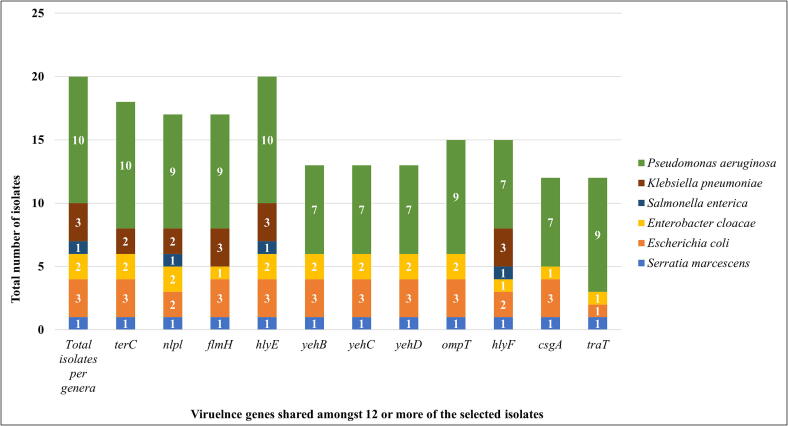
Most prevalent virulence genes, shared among the selected Enterobacterales and *Pseudomonas aeruginosa* strains isolated from smallholder farm environments.

**Figure 4:**
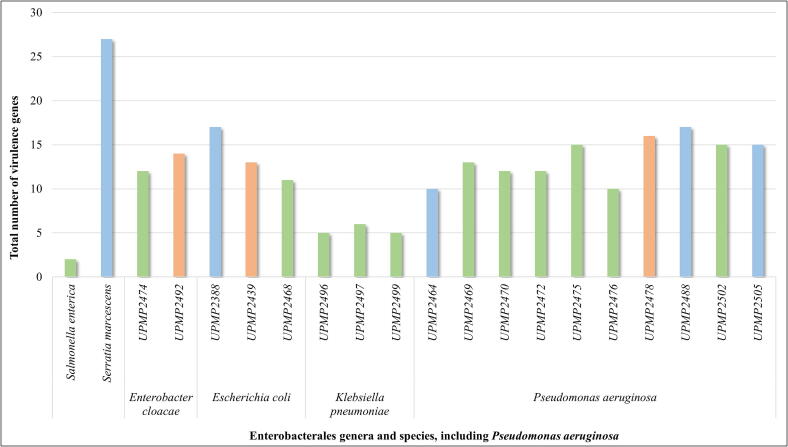
Total number of different virulence genes per isolate colored according to sample type (water – blue, soil – orange, fresh produce – green) from smallholder farms. (For interpretation of the references to colour in this figure legend, the reader is referred to the web version of this article.)

***In silico* analysis of virulence genes and sequence type (ST), as well as pathogenicity probability.** A total of 35 virulence genes were identified across the 20 isolates using VirulenceFinder. At least 11 genes were shared by 12 or more isolates (Fig. 3). The *hlyE* gene was present in all isolates, followed by *nlpl* in 90% (18/20), *terC* in 85% (17/20), and *flmH* in 80% (16/20) (Fig. 3). Other shared genes included the *yehBCD* complex in 65% (*n* = 13), *ompT* and *hlyF* in 75% (*n* = 15), *csgA* and *traT* in 60% (*n* = 12) (Fig. 3). The *S. marcescens* isolate from borehole water harbored the most genes (*n* = 27) (Fig. 4), followed by one *P. aeruginosa* (from dam water) and *E. coli* (from river water) with 17 different genes each (Fig. 4). The ten *Pseudomonas* isolates carried between 10 and 17 virulence genes, with *traT*, *terC*, *ompT*, *flmH*, *nlpl*, and *hlyE* commonly found across strains from soil, vegetables, and water sources (Figure 3, Figure 4).

Multilocus sequence typing (MLST) of the three *E. coli* isolates revealed distinct sequence types: ST48, ST254 and ST1141, isolated from borehole water (Farm C), baby carrots (Farm D), and soil (Farm F) (Table 3). The MLST analysis of the three *K. pneumoniae* isolates identified two STs: ST147 (two isolates from onions and bell peppers) and ST36, (isolated from spinach), all recovered from Farm F (Table 3). The two *E. cloacae* strains were identified with the same ST514, while four distinct STs (ST313, ST514, ST564, and ST3017) were identified among the *P. aeruginosa* isolates (*n* = 10) (Table 3). In-silico serotyping identified three *E. coli* serotypes (O45:H9, O113:H4, O156:H4) and distinct K-loci serotypes for *K. pneumoniae* (KL113:O4, KL178:O3a, KL113:O2a) (Table 3). Among the ten *P. aeruginosa* isolates, three serotypes were found (O1, O3, and O9) (Table 3).

**Table 3:** *In silico* multilocus sequence typing analysis, predicted serotypes and pathogenicity probability of selected Enterobacterales and *Pseudomonas aeruginosa*, isolated from water, soil, and fresh produce samples in South Africa

UPMP code	Identity	Source	Farm	Sequence type	Serotype	Pathogenicity probability	
						PathogenFinder	%
2457	*Serratia marcescens*	Borehole water	B	20	*	0,854	85
2388	*Escherichia coli*	Borehole water	C	48	O45:H9	0,835	84
2464	*Pseudomonas aeruginosa*	Borehole water	C	514	O3	0,752	75
2474	*Enterobacter cloacae*	Leeks	D	514	*	0,913	91
2468	*Escherichia coli*	Baby carrots	D	1141	O113:H4	0,931	93
2456	*Salmonella enterica*	Baby carrots	D	*	*	0,929	93
2469	*Pseudomonas aeruginosa*	Baby carrots	D	3017	O9	0,857	86
2470	*Pseudomonas aeruginosa*	Spring onions	D	313	O1	0,874	87
2472	*Pseudomonas aeruginosa*	Leeks	D	514	O3	0,751	75
2475	*Pseudomonas aeruginosa*	Rocket	D	514	O3	0,851	85
2476	*Pseudomonas aeruginosa*	Rocket	D	313	O1	0,826	83
2478	*Pseudomonas aeruginosa*	Soil	D	3017	O9	0,841	84
2488	*Pseudomonas aeruginosa*	Irrigation water	E	564	O9	0,847	85
2492	*Enterobacter cloacae*	Soil	F	514	*	0,933	93
2439	*Escherichia coli*	Soil	F	254	0156:H4	0,848	85
2496	*Klebsiella pneumoniae*	Onions	F	147	KL113:O4	0,803	80
2497	*Klebsiella pneumoniae*	Spinach	F	36	KL178:O3a	0,897	90
2499	*Klebsiella pneumoniae*	Bell pepper	F	147	KL113:O2a	0,901	90
2502	*Pseudomonas aeruginosa*	Municipal water	F	514	O3	0,842	84
2505	*Pseudomonas aeruginosa*	Onions	F	514	O3	0,842	84


* Unable to identify.

PathogenFinder was used to assess the potential similarity of the selected 20 isolates to human pathogens. The analysis predicted a similarity to human pathogens with confidence levels ranging from 0.835 to 0.931 for the *E. coli* strains (*n* = 3), 0.803 to 0.901 for the *K. pneumoniae* strains (*n* = 3), 0.913 to 0.933 for the *E. cloacae* strains (*n* = 2), 0.854 for the *S. marcescens* strain (*n* = 1), 0.929 for the *S. enterica* strain, and 0.751 to 0.874 for the *P. aeruginosa* strains (*n* = 10) (Table 3).

**Antimicrobial resistance and virulence genes in association with mobile genetic elements (MGEs).** Eight plasmid replicons were identified in the 10 Enterobacterales and 10 *P. aeruginosa* isolates, including pO111, the Col plasmid family (Col3M), and several Inc plasmid groups, such as IncFII, IncHI2, IncFIB, IncI, and IncX1 (Supplementary Table S3). The IncF group was found in four isolates and linked to multiple virulence genes (Supplementary Table S3). Specifically, IncFIC(FII) and IncFII were found in half (*n* = 2) of the IncF-positive isolates and were associated with *anr, traT,* and *traJ* virulence factors. The Col3M plasmid, linked to resistance genes qnrD1 and qnrS1 (encoding sulfonamide resistance), was detected in one *E. coli* isolate (borehole) and two *P. aeruginosa* isolates (fresh produce) (Supplementary Table S3). Lastly, the phage-like plasmid pO111 was detected in an *E. coli* isolate from chicken manure-amended soil and was associated with the *aph(3′)-la*, *blaTEM-176*, and *blaCTX-M-27* resistance genes (Supplementary Table S3).

A total of 23 insertion sequences (IS) linked to virulence and resistance genes were identified among the Enterobacterales and *P. aeruginosa* isolates (Supplementary Table S3). Notably, ISPa6 was found in 7/20 isolates and associated with the *fosA* resistance gene (Supplementary Table S3). Additionally, ISKpn19 associated with the *qnrS1* gene in various *E. cloacae, E. coli, S. marcescens,* and *P. aeruginosa* strains was identified (Supplementary Table S3). The β-lactamase gene *blaLAP-2* (associated with ISKpn19) was identified, along with ESBL genetic determinants *blaCTX-M-15* (associated with ISEc9) as well as *blaTEM-176* and *blaCTX-M-27* (associated with IS102) (Supplementary Table S3). Notably, two *P. aeruginosa* isolates from an onion sample and irrigation water shared identical MGEs: ISPa6, IS5075, and MITEEc1, along with their associated virulence and AMR genes (Supplementary Table S3).

## Discussion

To the authors’ knowledge, this is the first study to report on the prevalence, phenotypic and genotypic characterization of selected ESBL/AmpC-producing ESKAPE-E pathogens in the water-soil-plant nexus of smallholder farms in South Africa (SA). Overall, the findings align with global research indicating the presence of AMR genes and mobile genetic elements (MGEs) in Enterobacterales and Pseudomonads from environmental sources, posing potential public health risks (Zhu et al., 2024). The detection of MDR ESKAPE-E bacteria across water, soil, and fresh produce underscores the interconnectedness of agricultural environments as reservoirs of AMR, particularly in resource-limited smallholder farms. Building on a South African study by Richter et al. (2019) of ESBL/AmpC-producing Enterobacteriaceae in fresh produce from informal markets, the current study provides additional and novel insights into the production environment and farm practices. Moreover, these findings highlight key reservoirs of ARB in the overall spread of AMR within informal fresh produce supply chains.

The presence of multidrug resistant isolates found in water, soil, and fresh produce from four of the six farms aligns with reports for retailed fresh produce from SA (Richter et al., 2019) and Tunisia (Ben Said et al., 2016), suggesting that the informal supply chain may lack safeguards to control AMR contamination. Notably, irrigation sources including boreholes, river, and municipal water showed similar resistance profiles. Despite being limited to targeted water samples, this study confirms the extent of MDR bacteria in irrigation water in small-scale fresh produce, which aligns with previous research in Lebanon (Osman et al., 2024) and Ecuador (Montero et al., 2021). This emphasizes irrigation water as a conduit for introducing ARB and ARGs into agricultural systems, a pattern that is observed globally. Agricultural soils are known reservoirs for ARB, contributing to their spread along the food chain (Zhu et al., 2024). All soil isolates (*n* = 11) were resistant to aminoglycosides, cephalosporins, penicillins, and sulfonamides, mirroring 100% resistance patterns reported in Portugal (Amador et al., 2017) and Nigeria (Igbinosa et al., 2023). MDR is often driven by β-lactamases, including ESBLs and carbapenemases, and is compounded by additional resistance mechanisms (Noster et al., 2021).

Phenotypic confirmation of ESBL and/or AmpC production was observed in 56% and 50% of Enterobacterales isolates, differing from Richter et al. (2020) who reported 100% ESBL and 18.6% AmpC producers in commercial spinach supply chains in SA. The dominant ESBL producers included *K. pneumoniae* (*n* = 5), *E. cloacae* (*n* = 3), and *S. marcescens* (*n* = 2), in contrast to a study in Spain where *S. fonticola* was predominant (Pintor-Cora et al., 2021). The prevalence of presumptive ESBL-producing bacteria (33.4%) was higher than the 14.58% in commercial spinach production systems in SA (Richter et al., 2020) and 17.8% in vegetable farms in Spain (Pintor-Cora et al., 2021). This higher prevalence compared to commercial farms suggests unique risks within the informal supply chain, potentially including inadequate water quality or the proximity to livestock and/or waste. Among the *P. aeruginosa* isolates, 33.3% were ESBL- and 20% were AmpC-producers, higher than the 7.7% reported by Usui et al. (2019) in vegetables in Japan. Moreover, *P. aeruginosa*, an ESKAPE-E priority pathogen, is a major concern in healthcare-associated infections due to its resistance mechanisms and widespread prevalence (Denissen et al., 2022, World Health Organization (WHO), 2024a, World Health Organization, 2024b).

Colistin is an effective antimicrobial agent utilized against numerous clinically significant Gram-negative bacteria, including carbapenem-resistant *P. aeruginosa*, *K. pneumoniae*, and other Enterobacterales species (Hassen et al., 2022). Consistent with global trends, the mobile colistin resistance gene *mcr-9* was detected in one *S. marcescens* strain isolated from borehole water (Matteoli et al., 2021, Hassen et al., 2022). Although intrinsic colistin resistance in *S. marcescens* has been previously documented, it is notable that the *mcr-9* gene identified in this study was associated with the insertion sequence IS903. This aligns with findings by Hassen et al. (2022) in environmental and clinical isolates across 35 countries, where the association between *mcr-9* and IS903 suggests a potential mechanism for the gene’s mobilization both within and between bacterial species (Zheng et al., 2024). However, the inability of short-read sequencing in resolving long stretches of DNA prevents complete MGE assembly, thereby hindering our full understanding of the detected MGEs’ role in gene transfer among the environmental microbiota.

Across various farming environments and sample types, a wide range of bacterial species shared ARGs, particularly β-lactamases, underscoring the potential for ARG transfer between bacterial groups (Mitchell et al., 2024). Insertion sequences such as IS102, ISKpn19, and ISEc9, often linked to ESBL genes and resistance, were present in multiple isolates, facilitating ARG mobilization and promoting rapid MDR evolution (Zheng et al., 2024). For example, five isolates harboring *blaCTX-M-15*, including *K. pneumoniae* and *P. aeruginosa* strains isolated from fresh produce, coharbored ISEc9. In Northern Nigeria, ISEc9 was associated with *blaCTX-M-15* gene in hospitalized patients (Medugu et al., 2023), and in a recent study in KwaZulu Natal, SA, *K. pneumoniae* strains harboring ISEc9 were linked to the spread of *blaCTX-M-15* among patients (Hetsa et al., 2024). The cooccurrence of *blaCTX-M-15* with ISEc9 in *K. pneumoniae* and *P. aeruginosa* strains exemplifies how MGEs drive horizontal gene transfer (HGT), enabling rapid MDR evolution across human, animal, and environmental interfaces. The ability of MGEs to transfer resistance traits among ESKAPE-E pathogens reinforces the importance of surveillance systems to monitor and mitigate the spread of these resistant pathogens (Oyenuga et al., 2024).

In evaluating ARGs in potential ESKAPE-E pathogens, their spread across various environments was also assessed, for example, *P. aeruginosa* serotype O3 and ST514 (isolated from municipal water and the subsequently irrigated onions) has been reported in multiple environmental and clinical isolates across ten countries, including SA (Mihara et al., 2020, Kaszab et al., 2021, Nasrin et al., 2022). Furthermore, four virulence genes: namely *traT* (serum resistance), *csgA* (biofilm formation), *flmH* (adhesion proteins), and *terC* (oxidative stress tolerance) were shared among the *P. aeruginosa* strains, aligning with previous reports from butchering utensils in Iraq, and poultry environments in Egypt (Al-Kadmy et al., 2024, Rizk et al., 2024). The presence of virulence genes and globally disseminated STs in farm isolates underscores the potential for clinically relevant strains to circulate through One Health ecosystems, necessitating integrated surveillance.

The three *K. pneumoniae* strains sourced from fresh produce were identified with two sequence types: ST36, a hypervirulent clone linked to clinical isolates from Indonesia, Vietnam, and Australia (Holt et al., 2015, Feng et al., 2018), and ST147, a high-risk clone associated with infections in children across Northern Africa, India, Italy, and Greece (Peirano et al., 2020). The detection of ST147 in particular, a global MDR clone, in fresh produce highlights the permeability of boundaries between agricultural and clinical AMR transmission pathways. Likewise, three STs were identified among the *E. coli* strains—ST48 reported in poultry environments in Nigeria (Aworh et al., 2021), ST254 isolated from nonorganic lettuce in SA (Richter et al., 2024), and ST1141 sourced from animal feces, wastewater, and river water across China (Zheng et al., 2019). Furthermore, genes encoding iron acquisition (*sitA, iroN*), adhesins (*csgA, fdeC*), and enterotoxins (*astA, vat*) were detected among the *K. pneumoniae* strains, similar to previous reports from clinical and environmental samples in Romania and rural China (Chi et al., 2019, Surleac et al., 2020). These findings emphasize the potential role of smallholder farms as hubs for the exchange of hypervirulent clones between human, animal, and environmental interfaces – supporting a One Health approach to combating the spread of these pathogens.

## Conclusion

This study investigated the prevalence and dissemination of selected ESBL/AmpC-producing ESKAPE pathogens in South African smallholder farm environments. Key resistance and virulence genes, as well as associated mobile genetic elements, were identified, which underscores the role of fresh produce production environments as reservoirs and conduits for AMR pathogens that threaten public health. The presence of carbapenem and cephalosporin-resistant strains highlights the risk of antibiotic resistance gene transfer across agriculture, environment, and clinical sectors. Moreover, the observed prevalence of MDR Enterobacterales and *P. aeruginosa* strains, echoes global trends and indicates a need for integrated surveillance and intervention strategies to mitigate the spread of these pathogens.

## Data availability

The nucleotide sequences of the selected 10 Enterobacterales and 10 *P. aeruginosa* strains included in this study were deposited in the National Center for Biotechnology Information (NCBI) GenBank database in BioProject number: PRJNA642017.

## CRediT authorship contribution statement

**Sheldon Viviers:** Writing – review & editing, Writing – original draft, Visualization, Methodology, Investigation, Formal analysis, Data curation. **Loandi Richter-Mouton:** Writing – review & editing, Validation, Supervision, Project administration, Methodology, Investigation, Formal analysis, Data curation, Conceptualization. **Jonathan Featherston:** Methodology. **Lise Korsten:** Writing – review & editing, Supervision, Resources, Funding acquisition, Conceptualization.

## Funding

The authors would like to acknowledge the financial support of the Water Research Commission (WRC) funded project “Development of a fit-for-purpose water microbiological quality guideline for smallholder farmers and informal food traders” (WRC Proposal No 2022/2023-00885). The Department of Science and Innovation–National Research Foundation (DSI-NRF) Centre of Excellence in Food Security (Safe Food project ID 20301), and the 10.13039/100012534Centre for Environment, Fisheries and Aquaculture Science (Cefas) One Food project. MALDI-TOF analysis was based on the research supported in part by the NRF (UID 74426). Conclusions arrived at are those of the authors and are not necessarily to be attributed to the NRF. This study was also supported by the SEQAFRICA project which is funded by the Department of Health and Social Care’s Fleming Fund using United Kingdom (UK) aid. The views expressed in this publication are those of the authors and not necessarily those of the UK Department of Health and Social Care or its Management Agent.

## Declaration of competing interest

The authors declare that they have no known competing financial interests or personal relationships that could have appeared to influence the work reported in this paper.
