# Supplementary material for: Occurrence and Genotypic Characterization of Selected Multidrug-resistant ESKAPE-E Pathogens Isolated from Integrated Smallholder Fresh Produce Farms
Source: J Food Prot. 2025 Jun 23;88(7):100543. doi: 10.1016/j.jfp.2025.100543 (PMC12181083; doi:10.1016/j.jfp.2025.100543)
Supplement: Supplementary Data 1 [file mmc1.docx]

| **Supplementary Table S1:** Summary of the cultivation and production practices of the six smallholder farms sourced for antibiotic resistant bacteria in the current study | | | | | | |
| --- | --- | --- | --- | --- | --- | --- |
| **Site** | **Province** | **Scenario** | **Water source/s** | **Produce sampled** | **Irrigation method** | **Additional observations and information** |
| Farm A | Gauteng | Aquaculture | Borehole water | Kale, spinach and lettuce | Aquaponics and overhead irrigation (hosepipe) | Kale was grown in rocks |
|  |  |  | Aquaculture system used for nutrient enrichment before irrigation |  |  | Tilapia was produced as well |
| Farm B | Gauteng | Integrated | Borehole water | Kale, spinach and rape | Overhead irrigation (sprinklers) | Pig and chicken manure added directly to fields as fertiliser |
| Farm C | Gauteng | Organic | Borehole water | Lettuce, radishes and rocket | Overhead irrigation | Soil was amended with compost |
| Farm D | North West | Conventional river | River - canal system | Baby carrots, leeks, spring onions and rocket | Overhead irrigation | Supplied retailers and exported selected produce |
|  |  |  |  |  |  | GLOBAL'GAP certified |
| Farm E | Limpopo | Conventional dam | Dam - canal system | Tomatoes, green peppers and spring onions | Overhead irrigation (Tomatoes/spring onions) | Goats and sheep were also raised |
|  |  |  |  |  | Drip irrigation (green peppers) |  |
| Farm F | Gauteng | Conventional municipal | Municipal water | Spinach, green peppers and onions | Drip irrigation | Chicken manure was used to amend the fields |
|  |  |  |  |  |  | Liquid fertiliser was used in irrigation system |

| **Supplementary Table S2:** Antimicrobial resistance gene presence of the 20 selected ESKAPE-E isolates sourced from smallholder fresh produce environments | | | | | | | | | | | | | | |
| --- | --- | --- | --- | --- | --- | --- | --- | --- | --- | --- | --- | --- | --- | --- |
| **Isolate ID** | **Farm** | **Species** | **Source** | **Number of genes** | **Acquired antimicrobial resistance genes of different antimicrobial classes** | | | | | | | | | |
|  |  |  |  |  | **Aminoglycoside** | **Beta-lactam** | **Phenicol** | **Macrolide** | **Quinolones** | **Sulfonamide** | **Tetracycline** | **Fosfomycin** | **Formaldehyde** | **Colistin** |
| UPMP 2457 | B | *Serratia marcescens* | Borehole water | 9 | *-* | *bla_LAP-2_* | *-* | *-* | *qnrS1_1* | *-* | *tet(A)* | *fosA* | *formA* | *mcr-9_1* |
|  |  |  |  |  |  | *bla_CTX-M-14_* |  |  |  |  |  | *fosA3* |  |  |
|  |  |  |  |  |  | *bla_ACT-16_* |  |  |  |  |  |  |  |  |
| UPMP 2388 | C | *Escherichia coli* | Borehole water | 21 | *aadA2_1* | *bla_OXA-486_1_* | *catB7_1* | *mdf(A)_1* | *oqxA_1* | *dfrA17_1* | *tet(A)_6* | *fosA3_1* | *-* | *-* |
|  |  |  |  |  | *aadA5_1* | *bla_PAO_3_* | *cmlA1_1* |  | *qnrD1_1* | *sul2_2* |  | *fosA_4* |  |  |
|  |  |  |  |  | *ant(3")-Ia_1* |  | *crpP_1* |  | *qnrS1_1* | *sul3_2* |  |  |  |  |
|  |  |  |  |  | *aph(3")-lb_2* |  |  |  |  |  |  |  |  |  |
|  |  |  |  |  | *aph(3')-llb_2* |  |  |  |  |  |  |  |  |  |
|  |  |  |  |  | *aph(6)-Id_1* |  |  |  |  |  |  |  |  |  |
| UPMP 2474 | D | *Enterobacter cloacae* | Leeks | 8 | *aph(3')-la* | *bla_TEM-176_* | *floR* | *-* | *qnrS1* | *dfrA14* | *tet(A)* | *-* | *-* | *-* |
|  |  |  |  |  |  | *bla_CTX-M-27_* |  |  |  |  |  |  |  |  |
|  |  |  |  |  |  | *bla_ACT-12_* |  |  |  |  |  |  |  |  |
| UPMP 2456 | D | *Salmonella enterica* | Baby carrots | 2 | *aac(6')-laa* | *-* | *-* | *-* | *-* | *-* | *-* | *fosA7* | *-* | *-* |
| UPMP 2468 | D | *Escherichia coli* | Baby carrots | 8 | *aph(3')-la_3* | *bla_CTX-M-27_1_* | *floR_2* | *mdf(A)_1* | *qnrS1_1* | *dfrA14_5* | *tet(A)_6* | *-* | *formA* | *-* |
|  |  |  |  |  |  | *bla_TEM-176_1_* |  |  |  |  |  |  |  |  |
| UPMP 2492 | F | *Enterobacter cloacae* | Soil | 5 | *-* | *bla_CTX-M-14_* | *-* | *-* | *qnrS1* | *dfrA14* | *tet(A)* | *fosA3* | *-* | *-* |
|  |  |  |  |  |  |  |  |  |  |  |  |  |  |  |
| UPMP 2439 | F | *Escherichia coli* | Soil | 13 | *aph(3')-llb_2* | *bla_CTX-M-27_1_* | *catB7_1* | *mdf(A)_1* | *qnrS1_1* | *dfrA14_5* | *tet(A)_6* | *fosA_4* | *-* | *-* |
|  |  |  |  |  | *aph(3')-la_3* | *bla_OXA-488_1_* | *floR_2* |  |  |  |  |  |  |  |
|  |  |  |  |  |  | *bla_PAO_1_* |  |  |  |  |  |  |  |  |
|  |  |  |  |  |  | *bla_TEM-176_1_* |  |  |  |  |  |  |  |  |
| UPMP 2499 | F | *Klebsiella pneumoniae* | Onions | 13 | *aac(3)-IV* | *bla_CTX-M-15_* | *-* | *-* | *OqxB* | *sul1* | *tet(A)* | *fosA* | *-* | *-* |
|  |  |  |  |  | *aph(4)-la* | *bla_SHV-67_* |  |  | *OqxA* | *dfrA12* |  |  |  |  |
|  |  |  |  |  | *aadA2_1* | *bla_SHV-11_* |  |  | *qnrB2* |  |  |  |  |  |
| UPMP 2497 | F | *Klebsiella pneumoniae* | Spinach | 16 | *aph(6)-ld* | *bla_TEM-1B_* | *catB3* | *-* | *OqxB* | *sul2* | *tet(D)* | *-* | *-* | *-* |
|  |  |  |  |  | *aph(3")-lb* | *bla_OXA-1_* |  |  | *OqxA* | *dfrA14* |  |  |  |  |
|  |  |  |  |  | *aac(6')-lb-cr* | *bla_SHV-70_* |  |  |  |  |  |  |  |  |
|  |  |  |  |  | *aac(3)-lla* | *bla_SHV-11_* |  |  |  |  |  |  |  |  |
|  |  |  |  |  |  | *bla_SHV-13_* |  |  |  |  |  |  |  |  |
|  |  |  |  |  |  | *bla_CTX-M-15_* |  |  |  |  |  |  |  |  |
| UPMP 2496 | F | *Klebsiella pneumoniae* | Bell pepper | 16 | *aac(3)-IV* | *bla_PAO_* | *catB7_1* | *-* | *OqxB* | *sul1* | *tet(A)* | *fosA* | *-* | *-* |
|  |  |  |  |  | *aph(4)-la* | *bla_CTX-M-15_* |  |  | *OqxA* | *dfrA12* |  |  |  |  |
|  |  |  |  |  | *aph(3')-llb* | *bla_SHV-67_* |  |  | *qnrB2* |  |  |  |  |  |
|  |  |  |  |  |  | *bla_SHV-11_* |  |  |  |  |  |  |  |  |
|  |  |  |  |  |  | *bla_OXA-486_* |  |  |  |  |  |  |  |  |
| UPMP 2464 | C | *Pseudomonas aeruginosa* | Borehole water | 6 | *aph(3')-llb_2* | *bla_PAO_* | *catB7_1* | *-* | *-* | *-* | *-* | *fosA* | *-* | *-* |
|  |  |  |  |  |  | *bla_OXA-486_* | *crpP* |  |  |  |  |  |  |  |
|  |  |  |  |  |  |  |  |  |  |  |  |  |  |  |
|  |  |  |  |  |  |  |  |  |  |  |  |  |  |  |
|  |  |  |  |  |  |  |  |  |  |  |  |  |  |  |
| UPMP 2478 | D | *Pseudomonas aeruginosa* | Soil | 11 | *aph(3')-llb_2* | *bla_CTX-M-14_1_* | *catB7_1* | *mdf(A)_1* | *qnrS1_1* | *dfrA14_5* | *-* | *fosA3_1* | *-* | *-* |
|  |  |  |  |  |  | *bla_OXA-486_1_* |  |  |  |  |  | *fosA_4* |  |  |
|  |  |  |  |  |  | *bla_PAO_2_* |  |  |  |  |  |  |  |  |
| UPMP 2469 | D | *Pseudomonas aeruginosa* | Baby carrots | 7 | *aph(3')-llb_2* | *bla_OXA-486_1_* | *catB7_1* | *mdf(A)_1* | *-* | *-* | *tet(A)_6* | *fosA_4* | *-* | *-* |
|  |  |  |  |  |  | *bla_PAO_2_* |  |  |  |  |  |  |  |  |
| UPMP 2470 | D | *Pseudomonas aeruginosa* | Spring onions | 10 | *ant(3")-Ia_1* | *bla_CTX-M-14_1_* | *catB7_1* | *-* | *qnrD1_1* | *dfrA1_10* | *tet(A)_6* | *fosA3_1* | *-* | *-* |
|  |  |  |  |  |  | *bla_CTX-M-55_1_* |  |  |  | *sul1_5* |  |  |  |  |
|  |  |  |  |  |  | *bla_OXA-488_1_* |  |  |  |  |  |  |  |  |
| UPMP 2472 | D | *Pseudomonas aeruginosa* | Leeks | 5 | *aph(3')-llb_2* | *bla_PAO_* | *catB7_1* | *-* | *-* | *-* | *-* | *fosA* | *-* | *-* |
|  |  |  |  |  |  | *bla_OXA-486_* |  |  |  |  |  |  |  |  |
| UPMP 2476 | D | *Pseudomonas aeruginosa* | Rocket | 20 | *aac(3)-IIa_1* | *bla_CTX-M-15_1_* | *catB7_1* | *-* | *oqxA_1* | *dfrA14_5* | *tet(D)_1* | *fosA5_1* | *-* | *-* |
|  |  |  |  |  | *aac(6')-Ib-cr_1* | *bla_OXA-1_1_* | *crpP_1* |  | *oqxB_1* | *sul2_2* |  | *fosA_4* |  |  |
|  |  |  |  |  | *aph(3")-lb_5* | *bla_OXA-488_1_* |  |  |  |  |  |  |  |  |
|  |  |  |  |  | *aph(3')-llb_1* | *bla_PAO_4_* |  |  |  |  |  |  |  |  |
|  |  |  |  |  | *aph(6)-Id_1* | *bla_SHV-11_1_* |  |  |  |  |  |  |  |  |
|  |  |  |  |  |  | *bla_TEM-1B_1_* |  |  |  |  |  |  |  |  |
| UPMP 2475 | D | *Pseudomonas aeruginosa* | Rocket | 21 | *aac(3)-IVa_1* | *bla_CTX-M-15_1_* | *catB7_1* | *-* | *oqxA_1* | *dfrA12_8* | *tet(A)_6* | *fosA_4* | *-* | *-* |
|  |  |  |  |  | *aadA2_1* | *bla_OXA-486_1_* | *crpP_1* |  | *oqxB_1* | *sul1_5* |  | *fosA_5* |  |  |
|  |  |  |  |  | *aph(3')-llb_2* | *bla_PAO_3_* |  |  | *qnrB2_1* |  |  |  |  |  |
|  |  |  |  |  | *aph(4)-Ia_1* | *bla_SHV-11_1_* |  |  | *qnrD1_1* |  |  |  |  |  |
|  |  |  |  |  |  | *bla_TEM-1B_1_* |  |  | *qnrS1_1* |  |  |  |  |  |
|  |  |  |  |  |  |  |  |  |  |  |  |  |  |  |
|  |  |  |  |  |  |  |  |  |  |  |  |  |  |  |
| UPMP 2488 | E | *Pseudomonas aeruginosa* | Irrigation water | 9 | *aph(3')-llb_2* | *bla_OXA-485_1_* | *catB7_1* | *mdf(A)_1* | *qnrS1_1* | *sul1_5* | *-* | *fosA_4* | *-* | *-* |
|  |  |  |  |  |  | *bla_PAO_1_* |  |  |  |  |  |  |  |  |
|  |  |  |  |  |  | *bl_aTEM-1B_1_* |  |  |  |  |  |  |  |  |
| UPMP 2505 | F | *Pseudomonas aeruginosa* | Municipal water | 14 | *ant(3")-Ia_1* | *bla_CTX-M-14_1_* | *catB7_1* | *mdf(A)_1* | *-* | *dfrA1_10* | *tet(A)_6* | *fosA3_1* | *-* | *-* |
|  |  |  |  |  | *aph(3')-llb_2* | *bla_CTX-M-55_1_* | *crpP_1* |  |  | *sul1_5* |  | *fosA_4* |  |  |
|  |  |  |  |  |  | *bla_OXA-486_1_* |  |  |  |  |  |  |  |  |
|  |  |  |  |  |  | *bla_PAO_3_* |  |  |  |  |  |  |  |  |
| UPMP 2502 | F | *Pseudomonas aeruginosa* | Onions | 14 | *ant(3")-Ia_1* | *bla_CTX-M-14_1_* | *catB7_1* | *mdf(A)_1* | *-* | *dfrA1_10* | *tet(A)_6* | *fosA3_1* | *-* | *-* |
|  |  |  |  |  | *aph(3')-llb_2* | *bla_CTX-M-55_1_* | *crpP_1* |  |  | *sul1_5* |  | *fosA_4* |  |  |
|  |  |  |  |  |  | *bla_OXA-486_1_* |  |  |  |  |  |  |  |  |
|  |  |  |  |  |  | *bla_PAO_3_* |  |  |  |  |  |  |  |  |

| **Supplementary Table S3:** Mobile genetic elements associated with virulence and antimicrobial resistance genes in selected Enterobacterales and *Pseudomonas aeruginosa*, isolated from water, soil and fresh produce samples in South Africa | | | | | | | |
| --- | --- | --- | --- | --- | --- | --- | --- |
| **UPMP code** | **Identity** | **Source** | **Scenario** | **Mobile genetic elements (MGEs)** | | **Genes associated with MGEs** | |
|  |  |  |  | **Insertion sequence** | **Plasmid** | **Virulence** | **Resistance** |
| 2474 | *Enterobacter cloacae* | Leeks | Conventional/river | *IS102* |  |  | *aph(3')-la, blaTEM-176* |
|  |  |  |  | *IS102* | *po111* |  | *blaCTX-M-27* |
|  |  |  |  | *IS100* |  | *hha* |  |
|  |  |  |  | *ISEc38* |  | *hha* |  |
|  |  |  |  | *IS903* |  | *shiB* |  |
| 2492 | *Enterobacter cloacae* | Soil | Conventional/municipal | *ISKpn19* |  |  | *qnrS1, tet(A)* |
|  |  |  |  | *IS100* |  | *yehB, yehC, yehD, yehA* |  |
|  |  |  |  | *ISEc38* |  |  |  |
|  |  |  |  | *MITEEc1* |  | *terC, nlpl* |  |
|  |  |  |  | *ISEc1* |  | *fdeC* |  |
| 2388 | *Escherichia coli* | Borehole water | Organic | *ISPa6* |  |  | *fosA* |
|  |  |  |  | *ISVsa3* |  |  | *sul2* |
|  |  |  |  | *ISKpn19* |  |  | *qnrS1* |
|  |  |  |  |  | *Col3M* |  | *qnrS1* |
| 2439 | *Escherichia coli* | Soil | Conventional/municipal | *ISPa4, ISPa1* |  |  | *fosA* |
|  |  |  |  | *IS102* | *po111* |  | *aph(3')-la, blaTEM-176, blaCTX-M-27* |
| 2468 | *Escherichia coli* | Baby carrots | Conventional/river | *IS102* |  |  | *aph(3')-la, blaTEM-176, blaCTX-M-27* |
|  |  |  |  | *MITEEc1* |  | *ompT, terC* |  |
| 2496 | *Klebsiella pneumoniae* | Onions | Conventional/municipal | *ISPa6* |  |  | *fosA* |
|  |  |  |  | *ISEc59* |  |  | *aac(3)-IV, aph(4)-la* |
|  |  |  |  | *ISEc9* |  |  | *blaCTX-M-15* |
| 2497 | *Klebsiella pneumoniae* | Spinach | Conventional/municipal | *IS5075* |  |  | *sul2, aph(6)-ld, aph(3")-lb* |
|  |  |  |  | *IS6100* |  |  | *dfrA14* |
|  |  |  |  | *ISEc9* |  |  | *blaCTX-M-15* |
|  |  |  |  | *IS629* |  | *hha* |  |
| 2499 | *Klebsiella pneumoniae* | Bell pepper | Conventional/municipal | *ISEc59* |  |  | *aac(3)-IV, aph(4)-la* |
|  |  |  |  | *ISEc9* |  |  | *blaCTX-M-15* |
| 2457 | *Serratia marcescens* | Borehole water | Integrated | *ISKpn19* |  |  | *qnrS1, blaLAP-2, tet(A)* |
| 2913 | *Serratia marcescens* | Baby carrots | Conventional/river | *IS100* |  | *hha* |  |
|  |  |  |  | *ISEc38* |  | *hha* |  |
|  |  |  |  | *IS903* |  | *shiB* |  |
| 2456 | *Salmonella enterica* | Baby carrots | Conventional/river | *N/A* |  |  |  |
| 2464 | *Pseudomonas aeruginosa* | Borehole water | Organic | *ISPa6* |  |  | *fosA* |
| 2469 | *Pseudomonas aeruginosa* | Baby carrots | Conventional/river | *ISPa1* |  |  | *fosA* |
|  |  |  |  |  | *Incl1* |  | *tet(A)* |
|  |  |  |  | *ISEc31* |  | *nleB* |  |
|  |  |  |  | *MITEEc1* |  | *espA, tir, hha, eae-g02-theta, espF, nlpl, terC* |  |
|  |  |  |  | *IS609* |  | *yehB, yehD, yehC, yehA* |  |
| 2470 | *Pseudomonas aeruginosa* | Spring onions | Conventional/river |  | *Col3M* |  | *qnrD1* |
|  |  |  |  | *IS5075* |  |  | *tet(A)* |
| 2472 | *Pseudomonas aeruginosa* | Leeks | Conventional/river | *ISPa6* |  |  | *fosA* |
| 2475 | *Pseudomonas aeruginosa* | Rocket | Conventional/river | *ISPa6* |  |  | *fosA* |
|  |  |  |  |  | *IncX1* |  | *blaTEM-1B* |
|  |  |  |  | *ISEc59* |  |  | *aac(3)-IV, aph(4)-la* |
|  |  |  |  | *ISKpn19* |  |  | *qnrS1* |
|  |  |  |  |  | *Col3M* |  | *qnrD1* |
|  |  |  |  | *ISEc9* |  |  | *blaCTX-M-15* |
|  |  |  |  | *IS30* |  | *tsh* |  |
|  |  |  |  |  | *IncFIC(FII)* | *anr* |  |
|  |  |  |  |  |  |  |  |
|  |  |  |  |  |  |  |  |
|  |  |  |  |  |  |  |  |
|  |  |  |  |  |  |  |  |
|  |  |  |  |  |  |  |  |
| 2476 | *Pseudomonas aeruginosa* | Rocket | Conventional/river | *IS5075* |  |  | *blaTEM-1B, aph(6)-ld, aph(3")-lb, sul2* |
|  |  |  |  | *ISPa32* |  |  | *blaOXA-488* |
|  |  |  |  | *IS6100* |  |  | *dfrA14* |
|  |  |  |  | *ISEc9* |  |  | *blaCTX-M-15* |
|  |  |  |  |  | *IncFIB(AP001918)* | *ompT* |  |
|  |  |  |  | *IS629* |  | *hha* |  |
| 2478 | *Pseudomonas aeruginosa* | Soil | Conventional/river | *ISPa1* |  |  | *fosA* |
|  |  |  |  | *ISKpn19* |  |  | *qnrS1, tet(A)* |
|  |  |  |  | *IS100* |  | *yehB, yehA, yehD, yehC* |  |
|  |  |  |  | *ISEc38* |  |  |  |
|  |  |  |  | *ISEc81* |  |  |  |
|  |  |  |  | *MITEEc1* |  | *terC* |  |
|  |  |  |  | *ISEc32* |  | *traT, traJ* |  |
| 2488 | *Pseudomonas aeruginosa* | Irrigation water | Conventional/dam | *ISPa4, ISPa22, ISPa1* |  |  | *fosA, catB7* |
|  |  |  |  |  | *IncX1* |  | *qnrS1* |
|  |  |  |  | *Tn2, ISKpn19* |  |  | *blaTEM-1B* |
|  |  |  |  | *IS629* |  | *etsC, ompT, hlyF, ireA* |  |
|  |  |  |  | *MITEEc1* |  | *nlpl* |  |
|  |  |  |  |  | *IncFIC(FII)* | *anr* |  |
| 2502 | *Pseudomonas aeruginosa* | Municipal water | Conventional/municipal | *ISPa6* |  |  | *fosA* |
|  |  |  |  |  | *IncHI2A* |  | *dfrA1, aadA1, qacE, tet(A), sul1* |
|  |  |  |  | *IS5075* |  | *terC* |  |
|  |  |  |  |  | *IncFII* | *traT, traJ* |  |
|  |  |  |  | *ISEc5* |  | *gad* |  |
|  |  |  |  | *MITEEc1* |  | *terC* |  |
| 2505 | *Pseudomonas aeruginosa* | Onions | Conventional/municipal | *ISPa6* |  |  | *fosA* |
|  |  |  |  | *IS5075* | *IncHI2A* | *terC* | *dfrA1, aadA1, qacE, tet(A), sul1* |
|  |  |  |  |  | *IncFII* | *traJ, traT* |  |
|  |  |  |  | *MITEEc1* |  | *terC* |  |
